# Performance of gastrointestinal pathologists within a national digital review panel for Barrett’s oesophagus in the Netherlands: results of 80 prospective biopsy reviews

**DOI:** 10.1136/jclinpath-2020-206511

**Published:** 2020-05-28

**Authors:** Esther Klaver, Myrtle van der Wel, Lucas Duits, Roos Pouw, Kees Seldenrijk, Johan Offerhaus, Mike Visser, Fiebo ten Kate, Katharina Biermann, Lodewijk Brosens, Michael Doukas, Clément Huysentruyt, Arend Karrenbeld, Gursah Kats-Ugurlu, Jaap van der Laan, Ineke van Lijnschoten, Freek Moll, Ariadne Ooms, Jan Tijssen, Sybren Meijer, Jacques Bergman

**Affiliations:** 1 Department of Gastroenterology and Hepatology, Amsterdam UMC – Locatie AMC, Amsterdam, The Netherlands; 2 Department of Pathology, Amsterdam UMC – Locatie AMC, Amsterdam, The Netherlands; 3 Department of Pathology, Pathologie-DNA BV, Sint Antonius Hospital, Nieuwegein, The Netherlands; 4 Department of Pathology, UMC Utrecht, Utrecht, The Netherlands; 5 Department of Pathology, Symbiant BV, Alkmaar, The Netherlands; 6 Department of Pathology, Erasmus Medical Center, Rotterdam, Zuid-Holland, The Netherlands; 7 Department of Pathology, Stichting PAMM, Eindhoven, The Netherlands; 8 Department of Pathology, UMCG, Groningen, The Netherlands; 9 Department of Pathology, Haga Hospital, Den Haag, The Netherlands; 10 Department of Pathology, Isala Klinieken, Zwolle, The Netherlands; 11 Department of Pathology, Pathan BV, Sint Franciscus Vlietland Groep, Rotterdam, The Netherlands; 12 Department of Cardiology, Amsterdam UMC – Locatie AMC, Amsterdam, The Netherlands

**Keywords:** Barrett's oesophagus, digital pathology, quality assurance

## Abstract

**Aims:**

The histopathological diagnosis of low-grade dysplasia (LGD) in Barrett’s oesophagus (BO) is associated with poor interobserver agreement and guidelines dictate expert review. To facilitate nationwide expert review in the Netherlands, a web-based digital review panel has been set up, which currently consists of eight ‘core’ pathologists. The aim of this study was to evaluate if other pathologists from the Dutch BO expert centres qualify for the expert panel by assessing their performance in 80 consecutive LGD reviews submitted to the panel.

**Methods:**

Pathologists independently assessed digital slides in two phases. Both phases consisted of 40 cases, with a group discussion after phase I. For all cases, a previous consensus diagnosis made by five core pathologists was available, which was used as reference. The following criteria were used: (1) percentage of ‘indefinite for dysplasia’ diagnoses, (2) percentage agreement with consensus diagnosis and (3) proportion of cases with a consensus diagnosis of dysplasia underdiagnosed as non-dysplastic. Benchmarks were based on scores of the core pathologists.

**Results:**

After phase I, 1/7 pathologists met the benchmark score for all quality criteria, yet three pathologists only marginally failed the agreement with consensus diagnosis (score 68.3%, benchmark 69%). After a group discussion and phase II, 5/6 remaining aspirant panel members qualified with all scores within the benchmark range.

**Conclusions:**

The Dutch BO review panel now consists of 14 pathologists, who—after structured assessments and group discussions—can be considered homogeneous in their review of biopsies with LGD.

## Introduction

Low-grade dysplasia (LGD) is an important histological risk factor for progression to oesophageal adenocarcinoma in patients with Barrett’s oesophagus (BO). The histological diagnosis of LGD is however challenging, because early morphological changes of dysplasia are difficult to distinguish from reactive atypia of reflux-induced inflammation. As a result, the interobserver agreement for the diagnosis of LGD is poor, and LGD is widely overdiagnosed in community practice. Two studies from our group have shown that 73%–85% of the community LGD cases are downstaged to non-dysplastic BO (NDBO) by BO expert pathologists and that these downstaged cases have a low progression risk during follow-up. However, if the diagnosis LGD is confirmed by an expert BO pathologist, the risk of neoplastic progression is approximately 10% per patient-year.^1 2^ This risk increases significantly if multiple expert pathologists agree on this diagnosis.^3^

New guidelines dictate review of all dysplastic BO cases by an expert pathologist,^4–7^ but the concept of ‘expert pathologist’ is ill-defined, and access to expert review is not widely available. To facilitate expert review in the Netherlands, a national, web-based digital histology review platform has been set-up by the eight BO expert centres in the Netherlands. All diagnostic work-up and treatment of early BO neoplasia is centralised in these eight centres. The histology review panel was built around five ‘core pathologists’. These ‘core’ BO expert pathologists had a track record in the field of BO of >10 years (range 10–30 years), had participated in multiple teaching programmes (ie, www.best-academia.eu) and had each coauthored on >10 peer reviewed publications in this field.^8–12^

In a prior study, we evaluated if we could expand the number of pathologists in the panel to reduce the individual workload, reduce lead time and obtain nationwide coverage while maintaining the assessment quality and homogeneity.^12^ For this, 10 other gastrointestinal (GI) pathologists from the eight BO expert centres assessed digitalised slides of 60 endoscopy procedures, enriched for dysplasia. This case set had also been assessed by the five core pathologists. Three of the 10 pathologists met the stringent benchmark quality criteria for the case set, as established based on the results of the five core pathologists. Therefore, these three pathologists were considered to be homogeneous in their histological assessment with the five core pathologists and joined the core group of the expert panel. The majority of the other assessors showed only limited deviation from the preset benchmark scores. After this structured assessment, all pathologists participated in face-to-face group meetings where discrepant cases of the assessment rounds were discussed. Although we speculate that these assessments and group discussions will likely have improved their assessments and homogeneity, no formal evaluation has been performed on their performance.

Meanwhile, the national review panel has become operational. This platform is driven by reviews of the eight core pathologists and concentrates on revisions for alleged LGD and indefinite for dysplasia (IND) from centres throughout the Netherlands.

The aim of this study was to evaluate the performance of all pathologists of the eight BO expert centres in the assessment of the first 80 prospective LGD reviews submitted to the Dutch Barrett’s Pathology Review panel and to assess if the ‘non-core’ pathologists were homogeneous in their assessments with the eight core pathologists.

## Methods

### Case submission and scanning

Eighty BO surveillance cases with a diagnosis of IND or LGD during surveillance endoscopy were submitted to the Dutch Barrett’s Pathology Review panel (table 1).

**Table 1:** Patient and endoscopy characteristics

	Cases phase I (n=41)	Cases phase II (n=39)
Age at endoscopy in years (median, IQR)	67 (61–72)	66 (59–70)
Sex (male) (n, %)	26 (68)	31 (80%)
Levels biopsied (median, IQR)	2 (1–4)	2 (1–3)
Total biopsies (median, IQR)	8 (4–12)	7 (3–11)
Initial community diagnosis, n (%)		
IND	16 (39)	16 (41)
LGD	25 (61)	23 (59)

IND, indefinite for dysplasia; IQR, interquartile range; LGD, low-grade dysplasia.

Requests for consultation were submitted by gastroenterologists or pathologists via a dedicated website (www.barrett.nl), on which pathology slides were requested by the back office of the review panel. All slides were digitalised, using a scanner with a ×20 microscope objective (Slide, Olympus, Tokyo, Japan). Digitalised slides were checked for focus and acuity by the study coordinator, rescanned if necessary and stored on a secure server. Subsequently pathologists were invited by email to review the slides.

### Histological assessment

All 15 pathologists independently assessed the digital cases through a virtual slide system (Digital Slidebox 4.5, Leica Microsystems, Dublin, Ireland) allowing them to assess the slides similarly to their conventional microscopic diagnostic practice. Cases were scored according to the modified Vienna criteria for GI neoplasms.^13^ Diagnostic categories were: NDBO, LGD, high-grade dysplasia (HGD) or more, or IND.

The five initial core expert BO pathologists individually assessed all cases at an earlier stage. Group meetings were organised to discuss all cases in which their individual scores did not show a 4/5 or 5/5 agreement. As such a consensus diagnosis was generated as a reference for all pathologists’ individual assessments.

All pathologists assessed the first 41 cases in phase I, after which a group discussion was held to discuss discrepant cases. Cases were considered discrepant if three or more pathologists disagreed with the consensus diagnosis. The group discussion consisted of two sessions with one teleconference and one face-to-face meeting. The cases were shown on screen and discussed plenary. A total of nine cases was discussed. Pathologists had access to their own score and the consensus diagnosis after the group discussion but were unaware about their relative scores. After the group discussion, pathologists individually assessed another 39 cases in the phase II.

### Quality criteria

As described in our previous study,^12^ we used the following outcome parameters to quantify the quality of the individual histological assessment of each pathologist:

1. Percentage of diagnosis of IND per pathologist.
2. Percentage agreement with the consensus diagnosis per pathologist, using three diagnostic categories (NDBO, IND and LGD+HGD).
3. Percentage of cases with a consensus diagnosis of LGD or HGD underdiagnosed as NDBO per pathologist.

For these three quality criteria, we created benchmark scores based on the individual scores of the initial five core pathologists supplemented by the individual scores of the three pathologists who qualified as a core member based on their scores in the aforementioned study in which digitalised slides of 60 surveillance endoscopies were assessed.^12^

Benchmark ranges for each of the three criteria were based on a 95% prediction interval (PI) of the individual scores of these eight core pathologists. The 95% PI was calculated as the mean score from the eight pathologists ±2.365 times the SD (based on a t-distribution with 7 df, since n=8 pathologists). The upper or lower limit of these ranges, depending on the criterion, were used as benchmark values. Pathologists who met the benchmark score for all three quality criteria during phase I were added to the core, and benchmark scores for phase II were calculated based on the new extended core panel.

Anonymised materials were used in this study, hereby waiving the need for ethical approval by the medical ethical committee of the Amsterdam UMC or obtaining informed consent.

The statistical analyses were performed using the Statistical Package for the Social Sciences (SPSS V.24.0).

## Results

### Baseline characteristics of samples in case sets

Baseline characteristics of patients, endoscopic findings, number of biopsies obtained and original histological diagnoses of phase I and phase II were similar and are shown in table 1.

### Performance of pathologists in phase I

For the percentage of IND cases, six of seven aspirant panel members met the benchmark value of 28% with percentages ranging from 7.3% to 14.6%. Only one pathologist did not meet the required performance score and diagnosed 31.7% of cases as IND (table 2).

**Table 2:** Results of all pathologists for phase I (n=41)

Pathologist	Percentage of cases ‘indefinite for dysplasia’	Percentage agreement (three categories)*	Consensus LGD and HGD cases underdiagnosed as NDBO (n (%)) n=28
Core pathologists			
1	24.4	82.9	0
2	9.8	80.5	2 (7.1)
3	22.0	80.5	1 (3.6)
4	7.3	87.8	1 (3.6)
5	9.8	78.0	3 (10.7)
New core pathologists			
B	12.2	78.0	1 (3.6)
E	12.2	75.6	1 (3.6)
J	17.1	73.2	1 (3.6)
Aspirant panel members			
A	12.2	39.0	16 (57.0)
C	14.6	58.5	1 (3.6)
D	31.7	65.9	2 (7.1)
F	12.2	70.7	0
G	12.2	68.3	2 (7.1)
H	7.3	68.3	1 (3.6)
I	9.8	68.3	1 (3.6)
Benchmark value†	≤28	≥69	≤3 (11)

dary grey, score does not fall within benchmark values

light grey, score falls within benchmark values

*NDBO/IND/LGD+HGD.

†Based on eight core pathologists.

HGD, high-grade dysplasia; IND, indefinite for dysplasia; LGD, low-grade dysplasia; NDBO, non-dysplastic Barrett’s oesophagus.

For the percentage agreement with the consensus diagnosis, one pathologist met the benchmark lower limit of 69%, while three others just fell outside the range with a score of 68.3%.

The benchmark value for the percentage of consensus LGD and HGD cases underdiagnosed as NDBO was 11%, and six pathologists fell within this value. One pathologist clearly did not qualify for this criterion, underdiagnosing 16 of the 28 (57%) dysplasia cases as NDBO.

If all three quality criteria were taken into account, one pathologist met all benchmark scores and thus qualified as a core panel member. This pathologist was added to the core and incorporated in the benchmark ranges calculated for phase II. Four pathologists qualified for two criteria but showed a small deviation from the benchmark score for the percentage agreement with the consensus diagnosis. Two pathologists did not meet the required benchmark scores for two of the three quality criteria.

### Performance of pathologists in phase II

After the group discussion to discuss discrepant cases assessed in phase I and, subsequently, completing assessment of the next 39 cases, all pathologists fell within the benchmark score of ≤38% for the percentage of IND cases in phase II; scores ranged from 2.6% to 15.4% (table 3).

**Table 3:** Results of all pathologist for phase II (n=39)

Pathologist	Percentage of cases ‘indefinite for dysplasia’	Percentage agreement (three categories)*	Consensus LGD and HGD cases underdiagnosed as NDBO (n (%)) n=23
Core pathologists			
1	17.9	89.7	0
2	10.3	87.2	2 (8.7)
3	28.2	71.8	2 (8.7)
4	20.5	87.2	2 (8.7)
5	17.9	82.1	0
New core pathologists			
B	7.7	74.4	2 (8.7)
E	25.6	69.2	1 (4.3)
J	12.8	71.8	1 (4.3)
New core pathologist after phase 1			
F	33.3	61.5	1 (4.3)
Aspirant panel members			
A	2.6	56.4	7 (30.4)
C	7.7	66.7	0
D	12.8	64.1	0
G	12.8	66.7	2 (8.7)
H	12.8	69.2	1 (4.3)
I	15.4	76.9	1 (4.3)
Benchmarkvalue†	≤38	≥56	≤3 (13)

dark grey, score does not fall within benchmark values

light grey, score falls within benchmark values

*NDBO/IND/LGD+HGD.

†Based on nine core pathologists.

HGD, high-grade dysplasia; IND, indefinite for dysplasia; LGD, low-grade dysplasia; NDBO, non-dysplastic Barrett’s oesophagus.

All six remaining aspirant members fell within the benchmark range for percentage agreement with the consensus diagnosis.

Five of the six pathologists met the benchmark value for percentage of cases with a consensus diagnosis of LGD or HGD underdiagnosed as NDBO (table 3). They had zero (n=2), one (n=2) or two (n=1) underdiagnosed cases (median percentage 4.3% vs 4.3% for core pathologists).

One pathologist persisted in underdiagnosing a significant number of dysplastic cases as NDBO (7/23: 30.4%).

At the end of phase II, five of the six remaining aspirant panel members met all quality criteria.

## Discussion

The aim of this study was to evaluate the performance of the GI pathologists of the eight BO expert centres in the assessment of the first 80 consecutive review cases submitted to the Dutch Barrett’s Pathology Review panel with an initial diagnosis of IND or LGD. In our ambition to expand the current panel of eight core pathologists, while maintaining assessment quality and homogeneity, benchmark quality criteria based on the results of the core pathologists were used. These criteria enabled us to assess the ongoing process of homogenising future panel members. Assessments were done in two phases, with a group discussion to discuss discrepancies after phase I in order to further homogenise assessments.

When comparing the performance scores of the seven non-core pathologists to the benchmark scores, one out of seven aspirant panel members met all three benchmark quality criteria in phase I and was added to the core. Five out of the remaining six pathologists did not meet the benchmark value for only one of the three performance scores, percentage agreement, with three pathologists scoring only marginally below the required benchmark value (68.3% vs 69%). After the group discussion, five of the six remaining non-core pathologists met the benchmark values for all quality criteria during phase II.

This study is the third in a series of studies with this group of pathologists and includes the third independent set of histology cases. As with the previous studies, individual assessments were complemented with face-to-face group discussions, discussing discrepant cases from the slide set.^9–12^ The current study demonstrates that 14 pathologists can be considered homogenous in their assessments, which implies that these 14 pathologists are interchangeable as panel members of the Dutch Barrett’s Pathology Review panel.

In contrast to our earlier studies, we did not assess intraobserver agreement since the assessors only assessed all cases once. We decided not to use this variable as a benchmark quality criterion. Since kappa resembles the agreement percentage corrected for chance, kappa is influenced by the prevalence of the diagnosis and thus the variance over the different categories. Since the cases submitted for review by Dutch Barrett’s Pathology Review panel mainly consisted of dysplastic cases the variance is low, leading to a high agreement by chance. This results in low kappas and is therefore not a reliable representation of the quality of the assessments.

Strengths of this study are the use of a digitalised case set of consecutive BO cases with IND/LGD submitted for review from all over the Netherlands. Therefore, this case selection reflects the daily workload of the Dutch Barrett’s Pathology Review panel. This study builds on three earlier studies in which pathologists were trained through structured assessments and group discussions. All pathologists participating in this study come from the eight Barrett Expert Centers in the Netherlands and thus represent the full potential for histological reviews of the Dutch Barrett’s Pathology Review panel.

A limitation is that slides come from different laboratories around the Netherlands. This may have affected slide interpretability, especially for pathologists that are relatively new to reviewing cases from outside their own centre. However, this reflects the reality of our review panel, which per definition will receive consultations from different pathology laboratories.

Another point worth mentioning is that we used the consensus diagnosis made by our five core pathologists as training reference for the other pathologists. This means that our approach aimed to homogenise the assessments of the panel’s members. Our study set lacked follow-up information of the cases used and therefore we cannot prove that this homogenisation indeed improves outcomes. The consensus gold standard diagnosis in this study is based on five core pathologists who have extensive experience and an international reputation in the field of BO and have collaborated intensively in the Dutch Barrett advisory committee over the course of many years.^9 14 15^ Moreover, their assessments have been validated by comparing their diagnosis to the histological outcome during follow-up in multiple studies, demonstrating their diagnoses to be predictive of progression risk.^1–3^ Based on aforementioned data, we assume that the consensus diagnosis of our five core pathologists is a justified reference for the remaining panellists and that homogenising our panel based on this reference will likely improve outcomes.

In the future, several important steps will be taken while the Dutch Barrett’s Pathology Review panel proceeds.

A prediction model will be set up to establish how many pathologist’s assessments are needed in order to obtain a reliable diagnosis. The algorithm will incorporate the performance score of the pathologist in previous assessments with the outcome of his or her review to decide how many additional pathologists will have to review that specific case in order to retrieve a consensus diagnosis with the same reliability as used in the current study. This will enable an efficient and equal distribution of the workload.

Online group discussions will be continued to discuss cases without a majority diagnosis. In addition, annual trainings will be held, and assessment of homogeneity of all panel members will be renewed periodically.

This slide set of the first 80 consecutive review cases will be used in an online training programme for other pathologists and pathology residents to improve the histopathological assessment of BO. This training module will be accredited and freely available. Information from all study sets and group discussions will be incorporated. Pathologists and residents with an interest in BO will be able to improve their skills and compare their performance to our panel.

A lot of time and effort has been put in the training sets, assessment of digital pathology, development of the quality criteria and the infrastructure of the platform. Other future panels can benefit from these steps we have taken. In systems where endoscopic surveillance for Barrett’s patients is performed, there should be enough financial ability to run an expert panel since the panel will recoup its costs. Expert pathology will significantly improve the efficacy of Barrett surveillance by preventing unnecessary expensive endoscopies in patients with a low risk of progression and will also identify the subgroup with a truly increased risk of progression.

**Take home messages**

- Guidelines dictate expert review for a diagnosis of low-grade dysplasia in Barrett’s oesophagus (BO).
- A web-based digital expert review panel has been set up in the Netherlands.
- Pathologists from Dutch BO expert centres have participated in structured assessments and group discussions to homogenise assessments.
- This study shows that the expert review panel could be expanded to a total of 14 panel members while maintaining assessment quality and homogeneity by using predefined benchmark quality criteria for histological assessment of biopsies with low-grade dysplasia.
